# Annotations

**Published:** 1893-03-11

**Authors:** 


### Annotations.

**Catholicism and Physiology.** We are informed on good authority that physiology the physiology of the laboratory, is thoroughly taught at the Roman Catholic College of Oscott, near Birmingham, to the students who are preparing for the priestly life. The latest discoveries in physiological science are familiar to the teachers, and the fruits of experimental research are all laid under contribution, with the object of keeping the priestly intellect thoroughly in line with the general thought-currents of the age. In other words, the Catholic Church is determined that her clergy shall be as vividly alive in the modern as their predecessors have been in the ancient world. Now we are not of those who think that everything modern is new and true, and everything ancient childish and false. But for all that, the last two or three hundred years have made more extensive conquests of real knowledge than all the preceding ages put together. The mind, therefore, that does not live easily and familiarly in the present can scarcely be said to live at all. The fact that the Romish clergy are soundly instructed in physiology speaks volumes for the determination of the most ancient of the Churches to hold her own among the cultured and thoughtful of the future. This attitude on the part of the Roman priesthood is in striking contrast with the attitude of mind displayed by some of the prominent Anglican and Nonconforming clergy of our Protestant preference. Three hundred years ago the name "Protestant" connoted everything that was progressive, independent, and liberal in thought. What does it connote to-day among those who are not only for muzzling, but for defaming and degrading some of the most honourable and distinguished men of our generation, for mere difference of opinion and methods of work? For nobody denies the nobleness of the objects of those who devote their lives to the science of preventive medicine and physiological research. The objects are admitted to be beyond all praise. But if the objects are so noble, and the men so honourable, can the latter not be trusted to be the fittest judges of their own methods? We would ask Canon Wilberforce and other persons who have permitted themselves to be so overwhelmingly wrathful against physiologists to consider the example of Oscott. There are other ways of living the divine life than the way of religious observances; and the denouncers of physiologists would do well to consider whether, in speaking evil of those whose aim in life is as high as their own, but whose work and method are different, they are not sinning against the fundamental principles of their own faith.

**The Enthusiasm of Medicine.** Professor Seeley, in *Ecce Homo*, described what he called the "Enthusiasm of Humanity," and found in it the motive power which raised one human being to the highest intellectual and moral elevation to which any member of the human race had ever attained. The "enthusiasm of medicine" is now similarly doing for the healing art what has never been done for it before. At Oxford last week the Marquis of Salisbury insisted that medicine and her ancillary sciences constitute the modern antithesis to the ancient culture. The antithesis perhaps, but also the natural and inevitable complement when judged with sympathy, knowledge, and breadth of mind. This conception of medicine has not yet dawned upon the average medical intellect, much less upon the average non-medical mind. Nevertheless, it is the true conception, and the only conception which will give us a worthy and progressive medicine in the future. Medicine is, and will always remain, "the healing art"; but it is also, and is destined to become more and more, the discoverer of knowledge, the layer of new and deep foundations upon which to build human life and activity, the constructor of better, because truer, and really progressive philosophies. This, as Lord Salisbury insisted, is the true claim of modern scientific medicine to confidence and honour, that "She is sober, she is positive, she is absolute." "Positive and absolute" true medicine is, Lord Salisbury, himself a man of science, being the judge. But "positive and absolute" that medicine never can be which is merely the art of healing, and too often not even of healing but of pleasing or amusing fanciful idlers and hypochondriacs. The practising physician or surgeon, gathering in his gold, in too many instances knows little, and cares less, about the onward progress of a scientific, philosophical, and therefore eternally true and "positive medicine." Even the laboratory worker in the majority of cases works for facts, and facts alone, and thinks with contempt of the philosophical scientist who nobly endeavours to fit each fact into its appropriate place in the general scheme of the medical cosmos. But mere facts, though they be real as the rocks and innumerable as the stars, never can constitute a science, any more than millions of heaped and huddled bricks constitute a palace or a temple. There must be the seeing, the judging, and the constructive mind operating upon it all. The enthusiasm of the immediate past has given us the facts; we want now the ordering, the valuing, the clear and convincing exposition of the facts which shall turn the crude bricks of the laboratory into the scientific temple. In due time the noble enthusiasm of medicine will be the motive power which will give us this also.

**More Cholera Precautions?** The Port Sanitary Authority of the Port of London is keenly alive to the necessity of watchfulness as the spring approaches. Cholera hovers about the Continent like a dread spectre, and it may at any moment swoop down upon some doomed town, bringing ruin and destruction in its train. With such an enemy within a few hours' distance of us by sea it is of the utmost consequence that all ships entering the Port of London be scrutinised with the most searching scrutiny. There were issued on the 1st of March, to owners of vessels, certain specially enacted bye-laws which render it compulsory upon all ships' officers to notify to the medical officer of the port every case of dangerous infectious disease which they may happen to have on board, in order that the suffering and fever-spreading patients may be removed from the ships to the fever hospital at Gravesend or Greenwich. We assume that the special bye-laws are put in force with increased thoroughness at the present time because of the cholera alarm; though, oddly enough, whilst small-pox, diphtheria, erysipelas, scarlatina, typhus, typhoid, and other fevers are mentioned, not a single word is said on the subject of cholera. There may be reasons of a sufficient kind why this strange omission should have been made, but they do not suggest themselves spontaneously to the ordinary medical intelligence. No doubt cholera is included in "dangerous infectious diseases"; but inasmuch as there is no special mention made of it in the bye-laws, one might almost suppose they had been issued for the express purpose of leaving the Port of London open for cholera patients whilst visiting ships' officers with pains and penalties for failing to notify cases of fever of a less dangerous and much less infectious kind. Why, for example, notify typhoid and puerperal fever if cholera is to be specially excepted? We have looked most carefully through the bye-laws, even down to the signature "Henry H. Fowler," but have failed to find a single hint anywhere that cholera cases are to be notified as well as cases of other infectious diseases. The mystery is altogether too profound for us, and we give it up.

